# Effect of a Spinal Orthosis With Leaf Spring and Programmable Local Vibration on Kyphosis Angle and Trunk Muscle Strength in Hyperkyphosis: Development and Feasibility Study

**DOI:** 10.2196/82013

**Published:** 2026-06-25

**Authors:** Fatemeh Keshavarzi, Mokhtar Arazpour, Saeed Behzadipour

**Affiliations:** 1 Orthotics and Prosthetics Department, University of Social Welfare and Rehabilitation Sciences, Kodakyar St, Daneshjo Blvd, Evin, Tehran, 1985713871, Iran, 98 021-2218-0083, 98 021-2218-0109; 2 Djawad Movafaghian Research Center in Rehab Technologies, Sharif University of Technology, Tehran, Iran

**Keywords:** spine, orthotic devices, muscle strength, kyphosis, vibration

## Abstract

**Background:**

The application of spinal orthoses and local vibration in the treatment of hyperkyphosis is a developing concept. Further research is needed to determine the impact of leaf springs and local vibration techniques on the neuromusculoskeletal system.

**Objective:**

This study aimed to evaluate the effect of the simultaneous application of a leaf spring and a programmed local vibration system in a spinal orthosis on thoracic kyphosis angle (TKA) and the function of trunk flexor and extensor muscles.

**Methods:**

We used a soft thoracolumbar orthosis as a base for 2 parallel leaf spring bars made from AISI (American Iron and Steel Institute) 1075 steel (45 cm × 2 cm × 2 mm each). We added 4 solenoid actuators (60 g each) housed in 2 units positioned 12 cm apart, generating 20 N vertical force at 8 Hz frequency with 10 mm free displacement. A programmable Arduino microcontroller delivered 10-second vibration bursts followed by 5-minute rest intervals, repeated for 12 cycles per session, applied every other day. One participant with hyperkyphosis (baseline TKA=47.24°) used this device for 4 weeks. Outcomes included TKA (measured by photogrammetry) and trunk muscle function (assessed under isometric, isotonic, and isokinetic conditions). Feasibility outcomes included adherence, wear time, and adverse events.

**Results:**

Bench testing confirmed an 8 Hz vibration frequency, a 10 mm free displacement, and an on-body contact pressure of 9.5 N/cm² (95 kPa). In the short-term assessment (N=10), mean acceptability scores were 7.95 (SD 0.83) for appearance and 7.6 (SD 0.07) for comfort, and no skin-related adverse events occurred. In the 4-week single-participant pilot (N=1), adherence was 100% (12/12 sessions completed), mean daily wear time was 1.4 (SD 0.3) hours, and no adverse events were reported. TKA decreased from 47.24° to 45.81° (change: −1.43°), which did not exceed the minimal detectable change (4.62°). Pre-post changes in muscle function outcomes are reported descriptively.

**Conclusions:**

A semirigid thoracolumbar orthosis with integrated programmable vibration (8 Hz, 20 N free force) is technically feasible, showing reliable bench performance. Short-term assessment in 10 participants indicated acceptable comfort and appearance with no immediate safety concerns. The 4-week single-participant pilot demonstrated 100% adherence and no adverse events. The observed 1.43° kyphosis reduction was not clinically meaningful (below minimal detectable change). Larger controlled studies are needed to determine whether this device improves muscle function or spinal alignment.

## Introduction

Spinal orthoses have undergone significant evolution over decades, progressing from rigid immobilization devices to sophisticated soft exoskeletons designed to enhance functionality and patient outcomes [1]. Early iterations primarily used rigid structures to apply corrective or limiting forces, proving effective for postsurgical stabilization, injury protection, and the management of spinal deformities such as scoliosis and kyphosis [2]. However, the long-term adverse effects of rigid immobilization—including muscle atrophy and decreased bone mineral density—highlighted the need for alternative approaches [3]. This led to the development, by the late 1990s, of the first biomechanically informed orthoses incorporating semirigid and soft structures for kyphosis management [4].

Today, semirigid thoracolumbar orthoses are a standard conservative intervention for reducing thoracic kyphosis and improving muscle strength in postural and age-related hyperkyphosis [1]. While clinically effective, evidence suggests that they may not represent the most efficient conservative method for either kyphosis reduction or strengthening back musculature [5]. Consequently, orthotic design has continued to advance. A key development was the 2004 introduction of the Spinomed orthosis, which demonstrated improved outcomes [6]. Throughout this progression, the integration of leaf springs has been a notable feature, associated with significant clinical effectiveness [7,8]. Nonetheless, research has largely focused on therapeutic end points rather than on optimizing the material properties of the springs themselves [1].

Our previous work investigated leaf springs fabricated from AISI (American Iron and Steel Institute) 1075 carbon steel, a common and widely available material [8]. Although effective, the intervention produced a mean reduction in thoracic kyphosis of 8.14° (SD 10.5°), which was less than the 11.76° (10.4°) reduction achieved with the Spinomed orthosis [6]. Both orthotic interventions were, in turn, outperformed by supervised exercise training, as identified in a meta-analysis [5]. This performance gap underscores the need for further innovation in orthotic design.

To enhance the efficacy of semirigid thoracolumbar orthoses, we propose integrating a programmable local vibration system. Local vibration is a recognized intervention for mitigating muscle soreness and fatigue [9] and can promote muscle hypertrophy [10]. Although prior studies have explored vibration as a postural reminder in young populations with hyperkyphosis [11], its application for muscle strengthening in age-related hyperkyphosis—a condition strongly correlated with decreased back extensor strength [12]—remains underexplored. Commonly used displacement probes in muscular rehabilitation lack consistent evidential support [13], and concerns persist regarding potential muscle weakness from improper use. A programmable system could address these safety concerns, ensuring controlled application to protect muscle and skin integrity.

Spinal orthoses have evolved significantly from rigid, immobilizing braces toward semirigid and soft exoskeletons that aim to support function while mitigating the muscle atrophy and bone density loss associated with rigid designs [1,14,15]. While effective for stabilization, rigid structures often lead to discomfort and restricted mobility. Semirigid thoracolumbar orthoses now represent a standard conservative intervention for age-related hyperkyphosis, aiming to reduce the thoracic kyphosis angle (TKA) and improve back extensor strength [1]. Key innovations, such as the Spinomed orthosis and the integration of biomechanically tuned leaf springs, have enhanced corrective outcomes and trunk support [7,16]. However, despite these advancements, orthotic interventions generally remain less effective than supervised exercise programs, which meta-analyses have identified as producing superior kyphosis reduction through targeted strengthening [1,15-17].

Current orthotic research emphasizes clinical outcomes over material optimization [18], leaving potential for enhanced spring designs to close the efficacy gap with exercises [5]. Leaf spring materials for orthoses have been studied, with focus on optimizing flexibility, strength, and weight [7,8,16]. Common materials used in leaf springs include AISI 1075 carbon steel and composite polymers such as carbon fiber–reinforced plastics. Carbon fiber composites exhibit superior stiffness-to-weight ratios and fatigue resistance compared to traditional steel springs, leading to lighter orthoses with improved energy return and durability. Studies comparing steel and composite leaf springs report that composites exhibit up to ~30% higher stiffness and significantly lower stress under load, which may be beneficial for sustained corrective forces while reducing the risk of material fatigue. Thermoplastics with adjustable molding properties also allow customizable fit without compromising spring performance [19,20]. These material advances enable tunable mechanical properties in leaf springs for tailored spinal support devices [14].

Comparing the Spinomed orthosis versus exercise outcomes in hyperkyphosis, several randomized studies and meta-analyses find both interventions to be effective in reducing kyphosis angle and improving back extensor strength [21]. The Spinomed orthosis improves spinal alignment and muscle endurance by providing postural support and facilitating muscle activation, with kyphosis angle reductions reported between 7° and 12° after months of use [16,22]. However, recent randomized controlled trials of semirigid thoracolumbar orthoses in older adults with hyperkyphosis report improvements in TKA, trunk muscle strength, and balance after orthosis use for 6 to 12 weeks. One trial showed significant kyphosis reduction and increased isometric trunk extensor and flexor strength using a semirigid backpack-style orthosis compared to no treatment. Another randomized controlled trial demonstrated that Spinomed orthosis improved back muscle maximal voluntary contraction and balance parameters, highlighting muscle facilitation effects. However, trials underscore that orthoses often provide modest benefit relative to targeted exercise regimens, and none included programmable local vibration systems integrated with springs to date [8,16,23]. Meta-analyses highlight no significant difference between Spinomed orthosis and posture training support for immediate improvements, but supervised exercise tends to surpass orthoses in long-term outcomes [17]. Exercise programs, particularly those focusing on back extensor strengthening and postural training, often achieve greater kyphosis reductions and functional gains than orthoses alone, although adherence and safety vary [5,21].

Biomechanical analyses of leaf spring placement and force transmission in spinal supports emphasize critical design parameters, including spring position relative to spinal curvature, attachment points, and force vector alignment with anatomical axes. Optimal positioning on the posterior thoracolumbar region allows the application of assistive or resistive forces tailored to patient posture and muscle capacity. Modeling studies using finite element analysis and musculoskeletal simulations show that leaf springs modulate spinal loading by offloading vertebral structures and enhancing muscle activation through controlled resistance. Variations in spring material, thickness, and length affect stiffness and corrective moment magnitude, influencing comfort and clinical efficacy. These biomechanical insights guide the engineering of orthotic designs that dynamically interact with trunk biomechanics for improved kyphosis management [14,19,24]. Leaf springs function by selectively assisting or resisting trunk movements to offload bony and soft tissues or augment muscular performance. Their configuration—including positioning, attachment, material type, thickness, and length—dictates the magnitude and direction of the applied force, making them versatile for both exoskeletal and orthotic applications [14]. In exoskeletons, leaf springs are primarily protective, engineered to reduce lumbar loading during lifting or minimize strain during repetitive tasks [25]. They are typically offset from the body to align centers of rotation, generating corrective momentum while mitigating mechanical stress on anatomy and skin irritation from rigid contact [26,27]. In spinal orthoses, however, leaf springs are used chiefly to induce corrective spinal positioning. Positioned parallel and posterior to the spine, they apply controlled resistive or assistive forces directly to the torso to improve muscle function and, consequently, spinal alignment [6,8]. Unlike in exoskeletons, they often maintain direct body contact, with spacing adjustments used to modulate force or permit limited postural adjustment without rigid immobilization.

Whole-body vibration training and localized vibration systems have been shown to increase muscle mass and strength, counteracting sarcopenia and muscle fatigue. Randomized trials demonstrate that vibration frequencies around 60 Hz, with controlled amplitude, significantly enhance lower limb muscle activation and strength gains in seniors. Local vibration therapy, while established for muscle hypertrophy and fatigue reduction in sarcopenic older adults [28], remains underexplored in spinal orthoses for hyperkyphosis-related back extensor weakness. Prior applications focused on postural cues in young participants rather than strength gains in older individuals with hyperkyphosis [11,29,30]. Investigations integrating local vibration with spinal orthoses have primarily used parameters (eg, frequency, force amplitude) designed to enhance proprioception and postural awareness, rather than to induce muscular strengthening. To date, the application of local vibration within spinal supports has been limited primarily to serving as a postural reminder or neuromuscular stimulator in young, healthy participants [11,30]. Prior to the data derived from this study [31], no research on spinal orthotics had explicitly investigated local vibration with the primary goal of improving muscle strength. Using vibration parameters intended for strength adaptation in a wearable device, without a programmed and controlled application, carries a risk of adverse effects, including potential damage to skin and muscle tissues. This highlights the novelty of programmable vibration integration for safe, controlled muscle stimulation.

Programmable vibration devices allow safe, adjustable stimulation intensity addressing concerns about muscle or skin injury, making them a promising adjunct in geriatric orthopedic rehabilitation [31]. This study details the technical development and feasibility evaluation of a novel orthosis, based on the results from the first author’s doctoral research. It also specifically focuses on the technical specifications and development process of a novel semirigid thoracolumbar spinal orthosis equipped with 2 leaf springs and a programmable local vibration system, designed to reduce TKA and improve trunk muscle strength.

## Methods

### Study Design

**Figure 1.**
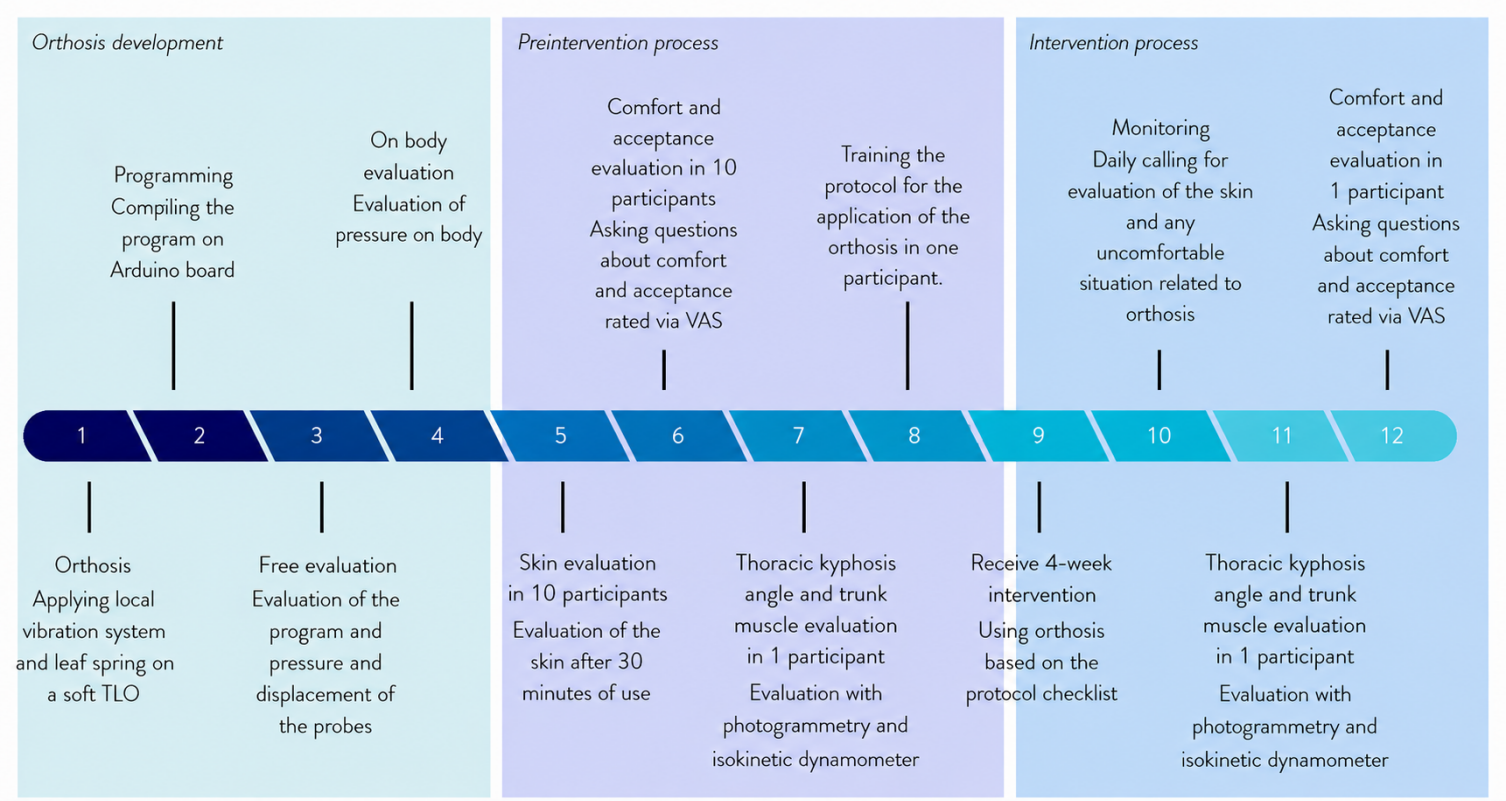
Implementation steps of the study including events in each section. TLO: thoracolumbar orthosis; VAS: visual analog scale.

This study comprised 3 sequential phases: (1) technical development and bench testing of the orthosis, (2) short-term (30 min) comfort, acceptability, and safety assessment in 10 participants with hyperkyphosis, and (3) a 4-week single-participant pilot to explore pre-post changes in TKA and trunk muscle function. The study was conducted in the orthotics and prosthetics department and the cumulative laboratory of the University of Social Welfare and Rehabilitation Sciences. To better understand the events, the implementation steps of the study are shown in Figure 1.

### Participants

#### Short-Term Assessment (Phase 2)

Ten participants with hyperkyphosis were enrolled to evaluate orthosis acceptability, comfort, and skin safety following a 30-minute wear trial. Participants were recruited from university faculties via email announcements.

#### Four-Week Single-Participant Pilot (Phase 3)

Following the short-term assessment, 1 participant proceeded to a 4-week usability trial.

#### Inclusion Criteria

The inclusion criteria for both phases are as follows: age 40 to 60 years, TKA>45° measured by photogrammetry, BMI of 25 to 33 kg/m², and the ability to walk independently without assistive devices while wearing the orthosis.

#### Exclusion Criteria

The exclusion criteria for both phases are as follows: osteoporosis (T-score <−2.5), recent osteoporotic vertebral fracture (within the past 6 mo), hyperkyphosis due to congenital or structural causes (eg, hemivertebra, Scheuermann disease), scoliosis (Cobb angle >10°), spinal canal stenosis, spinal tumors or infections, use of medications known to cause muscle weakness, diabetes with peripheral neuropathy or myopathy, and any spinal degenerative disease or neuropathic pain affecting the back or lower limbs [31].

### Phase 1: Technical Development and Bench Testing

#### Orthosis Development

**Figure 2.**
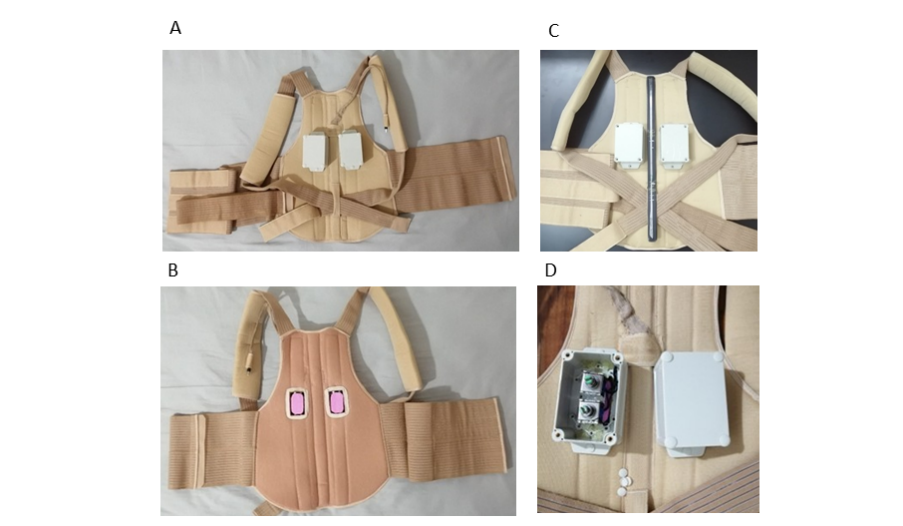
(A) and (B) The support structure. (C) The leaf spring position. (D) The placement of vibration units in the orthosis.

The orthosis consisted of a soft thoracolumbar support structure incorporating 2 parallel leaf springs fabricated from AISI 1075 steel (each 45 cm length × 2 cm width × 2 mm thickness). The support structure included 2 shoulder straps, each 70 cm in length, constructed from a dual-layer elastic fabric. The straps originated at the level of the seventh cervical vertebra (C7) and extended anteriorly and inferiorly over the shoulders, crossing each other posteriorly at approximately the level of the first lumbar vertebra (L1). They were then secured anteriorly over the abdomen via stitching to the lumbar elastic bands. The lumbar bands provided spinal coverage through a 2-step fastening system comprising wide elastic straps (Figure 2).

The leaf spring was custom-contoured to match the user’s thoracic and lumbar spinal curvature. In accordance with semirigid orthosis alignment protocols, the proximal portion was formed with a 4-cm offset from the spinal column. This spacing permitted an adequate range of motion for trunk movements while facilitating TKA reduction.

#### Local Vibration System

The vibration system included 4 solenoid actuators (60 g each) housed in 2 units (20 g each), positioned 12 cm apart vertically. Each solenoid generated 20 N vertical force at 8 Hz frequency with 10 mm free displacement. A programmable Arduino microcontroller delivered 10-second vibration bursts followed by 5-minute rest intervals, repeated for 12 cycles per session. Vibration was applied every other day (ie, 3‐4 sessions per week). The total electromechanical system mass was 300 g.

The vibration probes were strategically positioned over the paraspinal musculature between the T6 and T12 vertebral levels to target the erector spinae muscle group while avoiding direct vertebral column contact.

#### Battery and Thermal Protection System

The battery pack integrated a thermal fuse (72 °C, SD 3 °C), a battery management system (3 S, 10 A), and a polyfuse (10 A hold, 20 A trip) for safety. All electronic components (batteries, Arduino microcontroller, relays, and power switches) were housed in a separate portable control box, connected to the orthosis only during active vibration sessions. The solenoids were the only electronic elements permanently attached to the orthosis structure.

#### Bench Testing Measurements

##### Free Displacement and Vibration Frequency

The free displacement (amplitude) of the probes and the baseline vibration frequency (under no external load) were measured using high-speed video recording. Video playback was analyzed in slow motion to quantify oscillations per second (Hz).

##### Pressure

The pressure exerted by the vibration probes was measured under 2 conditions: (1) *free vibration*, with the system secured in a stabilizing box to maintain solenoid perpendicularity, and (2) *on-body vibration*, with the sensor placed between the orthosis-mounted probe and a healthy volunteer’s back. A 16×16 cm force-sensing resistor array integrated with the Pliance system (Novel GmbH) recorded pressure magnitude and distribution.

##### Weight

The total weight of the orthosis was measured, both with and without the integrated vibration system.

### Phase 2: Short-Term Comfort, Acceptability, and Safety Assessment (N=10)

Ten middle-aged participants with hyperkyphosis evaluated the orthosis appearance and comfort using 10-point numerical rating scales (0=completely unpleasant; 10=completely pleasant) for 2 items: (1) “Rate the orthosis’s appearance” and (2) “Rate the orthosis’s user-friendliness.” Each participant wore their size-matched orthosis for 30 minutes before providing feedback.

Skin safety was assessed via visual inspection [32] performed by a certified orthotist specializing in spinal orthoses. The evaluation was based on the presence of redness or abnormal skin color changes following a standardized fitting protocol. This protocol included an initial skin evaluation prior to orthosis application and a second evaluation after 30 minutes of wear [33]. Any observed erythema was expected to resolve within 20 minutes of orthosis removal. Persistent skin discoloration beyond this 20-minute period was recorded as an adverse event.

### Phase 3: Four-Week Single-Participant Pilot (N=1)

Following the short-term assessment, a single-participant pilot study was conducted. The participant (45-year-old woman, baseline TKA=47.24°) underwent a baseline assessment, orthosis fitting, and a 30-minute orientation session.

#### Intervention Protocol

Week 1 involved gradual adaptation, increasing wear time from 10 minutes. Weeks 2 to 4 involved maintenance wear of 1 to 2 hours daily (self-scheduled). Vibration (12 cycles of 10-s bursts with 5-min rests) was applied every other day. Throughout the intervention, the examiner made brief phone calls to monitor comfort and any adverse effects. The participant maintained a daily wear-time log.

#### Outcome Measures (Phase 3)

##### Thoracic Kyphosis Angle

TKA was assessed using photogrammetry, which demonstrates high test-retest reliability (intraclass correlation coefficient=0.97°; standard error of measurement=1.67°; minimal detectable change [MDC]=4.62°) [34,35]. The participant positioned herself on a marker while a calibrated camera (Canon 8 Mpixel MV150i; Canon Inc) captured 3 digital images of markers located on the seventh cervical and twelfth thoracic spinous processes [36]. Images were analyzed using AutoCAD (Autodesk, Inc) to compute angles, with the average of the 3 angles documented as TKA.

##### Trunk Flexor and Extensor Muscle Tests

Muscle function was assessed using a HUMAC-NORM isokinetic dynamometer (v.12.001.0005; CSMi) in the standing (orthostatic) position. System reliability in standing was 0.98 (Reyes-Ferrada et al, 2022). Table 1 summarizes the isometric, isotonic, and isokinetic test protocols.

**Table 1.** Summary of isometric, isotonic, and isokinetic test protocols.

Parameter	Isometric	Isotonic (eccentric or concentric)	Isokinetic concentric
Test mode	Isometric	Isotonic (constant load)	Isokinetic concentric
Test angle or ROM^a^	15° flexion	5° extension to 30° flexion	5° extension to 30° flexion
Speed	N/A^b^	Participant-controlled	60°/s
Evaluation torque	N/A	10 Nm	N/A
Warmup	Submaximal contractions (3 trials)	3 cycles familiarization	3 submaximal cycles
Trials	3 maximal per muscle group	3 cycles per mode	5 maximal repetitions
Rest intervals	30 seconds between trials; 1 minute between groups	30 seconds between trials	30 seconds between trials
Visual feedback	Off	Off	Off
Key variables	Peak torque, average torque, time to peak torque	Peak power, work per repetition, joint angle at peak power	Peak torque, work per repetition, time to peak torque

a ROM: range of motion.

b N/A: not applicable.

##### Feasibility Outcomes

Feasibility outcomes were assessed using descriptive statistics: adherence (percentage of prescribed sessions completed), wear time (minutes per day; mean, SD), device issues (frequency and type), and safety (number and severity of adverse events).

##### Pain and Usability Assessment

The participant rated ease of wearing, ease of use, weight, and appearance using 0 to 10 rating scales postintervention.

### Statistical Analysis

#### Study Design and Data Structure

This feasibility study used a single-participant pre-post design. The same participant underwent outcome assessments before and after a 4-week intervention, yielding paired observations for each outcome measure.

#### Clinically Meaningful Change Thresholds

A priori decision rules were established to determine whether observed changes were clinically meaningful. Thresholds were derived from published MDC values and percentage change criteria from orthosis and vibration literature [16,31,34].

#### Feasibility Outcomes

Feasibility outcomes were assessed using descriptive statistics: adherence (percentage of prescribed sessions completed), wear time (minutes per day; mean, SD), device issues (frequency and type), and safety (number and severity of adverse events).

### Ethical Considerations

This study was approved by the Ethics Committee of the University of Social Welfare and Rehabilitation Sciences (approval: IR.USWR.REC.1401.217) in January 2023.

## Results

### Phase 1: Technical Specifications and Bench Testing Performance

The electromechanical vibration module affixed to the orthosis had a total mass of 300 g, comprising 2 housing units (20 g each) and 4 solenoid actuators (60 g each).

#### Vibration Frequency

An 8 Hz vibration frequency was verified using video recording. Footage of the active module was analyzed using DaVinci Resolve Studio software (v.20.0.1.6; Blackmagic Design Pty Ltd). The analysis confirmed that the probe achieved its maximum displacement from the solenoid body 8 times per second, corresponding to the target 8 Hz frequency.

#### Mechanical Pressure

Pressure was quantified using a Pliance system (Novel GmbH). The peak force measured in the free-state was 251.2 N. Given the circular probe’s cross-sectional area of 12.56 cm², this equated to a contact pressure of 20.0 N/cm² (200 kPa). During on-body use, the peak force decreased to 119.3 N, resulting in a measured contact pressure of 9.5 N/cm² (95 kPa).

#### Probe’s Free Displacement

The maximum linear displacement of the solenoid probe was measured as 1.0 cm from its resting position.

### Phase 2: Short-Term Comfort, Acceptability, and Safety Assessment (N=10)

Ten participants with hyperkyphosis evaluated the orthosis. Their mean age was 62.2 (SD 7.83) years, and their mean TKA was 55.53° (SD 1.69°). The average score for appearance was 7.95 (SD 0.83), and the average score for comfort was 7.6 (SD 0.07). Detailed demographic data and individual responses are reported in Table 2. No skin-related adverse events were observed following the 30-minute wear trial.

**Table 2.** Demographic data and feedback from phase 2 participants (N=10).

Participants	Age, y	Gender	Height, m	Weight, kg	TKA^a^	Appearance score	Comfort score
1	64	Woman	1.70	69.00	49.31°	9.5	8
2	60	Woman	1.63	60.00	56.89°	9	8
3	60	Woman	1.65	67.00	56.07°	8	8
4^b^	62	Woman	1.57	55.00	61.26°	8	7
5	62	Woman	1.52	58.00	57.41°	8	8
6	62	Woman	1.49	66.00	53.33°	8	6
7	63	Woman	1.55	69.00	50.41°	7	8
8	67	Woman	1.50	70.00	63.25°	8	8
9^c^	77	Man	1.76	90.00	60.11°	7	7
10	45	Woman	1.50	51.00	47.24°	7	8
Mean (SD)	62.2 (7.83)	—^d^	1.59 (0.09)	65.5 (10.82)	55.53° (1.69°)	7.95 (0.83)	7.6 (0.07)

a TKA: thoracic kyphosis angle.

b Mentioned unpleasant characteristics: heavy.

c Mentioned unpleasant characteristics: hard to wear.

d Not applicable.

### Phase 3: Four-Week Single-Participant Pilot—Pre-Post Changes

#### Feasibility and Adherence Outcomes

A 45-year-old woman with hyperkyphosis used the orthosis with the local vibration system every other day for 4 weeks. Feasibility and adherence outcomes are summarized in Table 3.

**Table 3.** Feasibility and adherence outcomes.

Feasibility metric	Result	Target	Whether target met
Adherence			
Prescribed sessions (total)	12	12	Yes (100%)
Completed sessions	12	12	Yes (100%)
Missed sessions	0	0	Yes
Wear time (h)			
Prescribed daily wear	1‐2	1‐2	Yes
Actual mean (SD) daily wear	1.4 (0.3)	1‐2	Yes
Total wear time over 4 weeks	39.2	28‐56	Yes
Device issues			
Technical failures	0	0	Yes
Battery depletion during session	0	0	Yes
Skin adverse events	0	0	Yes
Reported discomfort (0‐10 scale)	2/10	<4/10	Yes
Acceptability (postintervention; 0‐10 scale)			
Ease of wearing	8	≥7	Yes
Ease of use	7	≥7	Yes
Weight acceptance	7	≥6	Yes
Appearance	5	≥5	Yes (borderline)

#### Thoracic Kyphosis Angle

TKA was 47.24° preintervention and 45.81° postintervention (change: −1.43°). This change did not exceed the MDC value of 4.62° reported for photogrammetry [34], indicating that the observed reduction was not clinically meaningful.

#### Trunk Muscle Function: Pre-Post Changes

Preintervention and postintervention values for all muscle function outcomes are described in Table 3. Isometric extensor peak torque increased from 19 to 24 Nm (+5 Nm). Isometric flexor peak torque decreased from 34 to 25 Nm (−9 Nm). Isokinetic concentric flexor peak torque increased from 6 to 20 Nm (+14 Nm). Isokinetic concentric extensor peak torque increased from 12 to 20 Nm (+8 Nm). Complete descriptive results for isometric, isotonic, and isokinetic tests are presented in Table 4.

**Table 4.** Decision rules for clinically meaningful change.

Outcome	Threshold type	Threshold value	Observed change	Whether threshold met	Decision
TKA^a^ reduction	MDC^b^	≥4.62°	1.43°	No	Not clinically meaningful
Isometric extensor torque	% change	≥15%	+26.3%	Yes	Clinically meaningful
Isokinetic extensor torque	% change	≥20%	+66.7%	Yes	Clinically meaningful
Isokinetic flexor torque	% change	≥20%	+233%	Yes	Clinically meaningful
Time to peak torque	% reduction	≥15%	−55.2%	Yes	Clinically meaningful
Adherence to protocol	% completion	≥80%	100%	Yes	Feasible
Adverse events	Count	0	0	Yes	Safe

a TKA: thoracic kyphosis angle.

b MDC: minimal detectable change.

#### Pain and Usability Assessment

The participant assigned the orthosis scores of 8 out of 10 for ease of wearing, 7 out of 10 for ease of use, 7 out of 10 for weight, and 5 out of 10 for appearance. No adverse events were reported during the 4-week intervention.

## Discussion

### Overview

This technical note describes the development and feasibility evaluation of a semirigid thoracolumbar orthosis integrating a programmable local vibration system. The study had 3 phases: (1) bench testing confirming technical specifications (8 Hz, 10-mm free displacement, 9.5 N/cm² on-body contact pressure); (2) short-term assessment in 10 participants showing acceptable comfort (7.6 out of 10) and appearance (7.95 out of 10) with no skin safety concerns; and (3) a 4-week single-participant pilot demonstrating 100% adherence, no adverse events, and descriptive pre-post changes in kyphosis angle and muscle function outcomes.

### Technical Feasibility

The bench testing confirmed that the electromechanical system met its design specifications. The 8-Hz vibration frequency was reliably achieved, and the on-body contact pressure of 9.5 N/cm² (95 kPa) was lower than the free-state pressure (20.0 N/cm²) due to tissue compliance and padding. This reduction is consistent with previous reports of wearable vibration devices [32] and suggests that the system delivers mechanical stimulation within a range that is likely safe for soft tissue, though formal tissue tolerance studies would be needed to confirm.

The choice of 8 Hz frequency warrants discussion. While higher frequencies (30‐60 Hz) are often used for proprioceptive training [37,38], lower frequencies (8‐15 Hz) have been shown to improve muscle strength with potentially lower risk of tissue irritation [39,40]. Since participants in this study received vibration during seated isometric postural holding, 8 Hz was selected as a conservative starting point that balances potential neuromuscular effects with safety. The 10-second vibration bursts with 5-minute rest intervals were based on neurophysiological evidence indicating that muscle spindle afferents require approximately 6 seconds to return to baseline activity following mechanical stimulation [41].

### Short-Term Acceptability and Safety (Phase 2)

The short-term assessment in 10 participants with hyperkyphosis revealed acceptable scores for appearance (7.95/10) and comfort (7.6/10). Among these participants, 3 noted specific concerns: 1 reported the orthosis was “heavy,” 1 reported it was “hard to wear,” and 1 reported no specific concerns. No skin-related adverse events were observed after 30 minutes of wear. These findings suggest that the orthosis is acceptable for short-term use, though the “hard to wear” comment highlights the need for improved donning instructions or design modifications.

### Four-Week Single-Participant Pilot (Phase 3)

#### Feasibility

The participant completed all prescribed sessions with a mean daily wear time of 1.4 (SD 0.3) hours, meeting the prescribed target of 1 to 2 hours. No technical failures, battery depletions, or adverse events occurred. These feasibility metrics support the safe application of the device for home use in future larger studies.

#### Thoracic Kyphosis Angle

The observed reduction of 1.43° (from 47.24° to 45.81°) did not exceed the MDC=4.62° established for photogrammetry [34]. Therefore, this change cannot be interpreted as a clinically meaningful improvement. Possible explanations for the modest change include the short intervention duration (4 wk) or the mild baseline kyphosis (<52°). Future studies with longer intervention periods and participants with more severe kyphosis may be needed to detect meaningful spinal alignment changes.

#### Muscle Function Outcomes Based on Descriptive Findings

Pre-post changes in muscle function are reported descriptively (Table 3) and should be interpreted with caution, given the single-participant design. Several observations are noted, including the following: isometric extensor peak torque increased by 5 Nm (from 19 to 24 Nm), whereas isometric flexor peak torque decreased by 9 Nm (from 34 to 25 Nm). Isokinetic concentric peak torque increased for both flexors (from 6 to 20 Nm) and extensors (from 12 to 20 Nm). Time-to-peak torque decreased for both flexors and extensors across several test types.

While these descriptive changes are notable, they cannot be attributed to the intervention with certainty due to the absence of a control group, repeated measures, or inferential statistics. The patterns observed may reflect true neuromuscular changes, practice effects, measurement variability, or a combination of factors. Importantly, no electromyography or mechanistic measurements were collected; therefore, proposed mechanisms such as altered cocontraction, reciprocal inhibition, or motor unit recruitment remain speculative hypotheses requiring direct investigation in future studies.

Previous studies of local vibration in spinal orthoses have primarily used vibration as a postural reminder (eg, 30‐60 Hz vibration motors) rather than as a therapeutic stimulus for muscle strengthening [11,29,30]. These studies reported reductions in kyphosis angle of approximately 8° after 4 days of use [30], which exceeds the change observed in this study. The difference may be due to the younger population studied (mean age 28.06, SD 6.06 y), the type of vibration (high-frequency, low-amplitude vibration motors vs 8 Hz solenoid drivers), or the vibration protocol (continuous vs intermittent).

In contrast, this study used a lower frequency (8 Hz) with higher force (20 N free, 9.5 N worn) delivered intermittently (10 s on, 5 min off). This protocol was designed to prioritize safety for older adults and to avoid potential overstimulation. The trade-off may be a slower onset of therapeutic effects, which could require longer intervention periods to detect meaningful changes.

This feasibility study provides the technical foundation for the trial and demonstrates that the device is safe and usable before proceeding to larger controlled studies. Based on descriptive results, we hypothesized mechanisms that require future investigation. The following mechanisms are speculative hypotheses derived from the descriptive findings. They were not directly measured in this study and hence require confirmation through electromyography, neurophysiological, or mechanistic studies. The descriptive pattern of muscle function changes—increased isometric extensor torque but decreased isometric flexor torque, alongside increased isokinetic torque in both muscle groups—might be explained by several hypothetical mechanisms:

1. Reduced antagonistic cocontraction: a decrease in flexor torque during isometric testing might indicate reduced cocontraction of antagonist muscles, which could improve postural efficiency and reduce spinal compressive loads [42]. However, without electromyography data, this remains speculative.
2. Task-specific neuromuscular adaptation: the contrasting results between isometric (flexor torque decrease) and isokinetic (flexor torque increase) tests might suggest that the intervention affects motor control strategies differently depending on the contraction type and speed [43]. This hypothesis requires direct testing.
3. Vibration-induced neural effects: local vibration is known to influence muscle spindle afferents and may alter cortical and spinal excitability [44,45]. These effects could theoretically lead to improved motor unit recruitment efficiency, as suggested by a decreased time to peak torque [46]. However, no neurophysiological measurements were collected in this study.

Future studies should incorporate surface electromyography to quantify agonist-antagonist activation ratios, cocontraction indices, and recruitment patterns during functional tasks. Mechanistic studies using transcranial magnetic stimulation or H-reflex testing are needed to confirm central versus peripheral adaptations.

### Limitations

This study has several important limitations as discussed in the following

- Single-participant pilot (phase 3): Only 1 participant completed the 4-week intervention. Therefore, the findings cannot be generalized to the broader population of individuals with hyperkyphosis. Pre-post changes are reported descriptively without inferential statistics (no *P* values, CIs, or effect sizes), as such tests are not valid for N=1.
- No control group: The absence of a control or comparison group means that observed pre-post changes cannot be attributed to the intervention with certainty. Practice effects, natural history, placebo effects, or measurement variability may account for some or all of the observed changes.
- Short intervention duration: Four weeks may be insufficient to detect meaningful changes in spinal alignment, particularly in individuals with mild baseline kyphosis (47.24°). Longer intervention periods (eg, 12‐24 wk) may be needed for structural spinal changes.
- No mechanistic measurements: electromyography, neurophysiological (H-reflex, transcranial magnetic stimulation), or imaging studies were not conducted. Therefore, the proposed mechanisms involving cocontraction, motor unit recruitment, or neural adaptation remain speculative and should be interpreted as hypotheses requiring future testing.
- Modest kyphosis change: The 1.43° reduction in TKA did not exceed the MDC (4.62°), indicating that this change was not clinically meaningful. This finding should not be interpreted as evidence of clinical effectiveness.
- Single site, single orthotist: All assessments and fittings were conducted by the same research team at one institution, which may limit reproducibility and generalizability.
- Participant selection: Phase 2 participants (N=10) had a mean age of 62.2 (SD 7.83; range 45‐77) years and a mean kyphosis of 55.53° (SD 1.69°). The phase 3 participant was 45 years old with milder kyphosis (47.24°), limiting the applicability to older adults or those with more severe hyperkyphosis. Outcomes were assessed immediately after the 4-week intervention. The durability of any observed changes and long-term safety remain unknown.

### Clinical Implications (Preliminary)

At present, this device should be considered an investigational tool rather than a clinically proven intervention. Based on this feasibility study alone, the orthosis cannot be recommended for clinical use to improve kyphosis angle or trunk muscle strength. The primary contributions of this study are as follows: (1) demonstrating that a programmable vibration system can be successfully integrated into a semirigid spinal orthosis, (2) providing bench-tested technical specifications, and (3) establishing preliminary safety and adherence data to justify larger controlled trials. Clinicians and researchers may use these findings to guide the design of future studies but should not interpret the descriptive pre-post changes as evidence of clinical effectiveness.

### Conclusion

A semirigid thoracolumbar orthosis with integrated programmable vibration (8 Hz, 20 N free force, 9.5 N/cm² on-body contact pressure) was successfully developed and tested. Bench testing confirmed reliable technical performance. Short-term assessment in 10 participants with hyperkyphosis indicated acceptable comfort and appearance (scores 7.6‐7.95 out of 10), with no immediate skin safety concerns. In a 4-week single-participant pilot, adherence was 100%, daily wear time met the prescribed target (mean 1.4, SD 0.3 h), and no adverse events were reported. The observed 1.43° reduction in TKA did not exceed the MDC (4.62°) and, therefore, was not clinically meaningful. Descriptive pre-post changes in muscle function outcomes (eg, isokinetic peak torque increases of 8‐14 Nm) are reported but cannot be attributed to the intervention without a control group or inferential statistics. This study demonstrates technical feasibility and safety, justifying progression to larger controlled studies with adequate sample sizes, control groups, and mechanistic measurements (eg, electromyography) to determine whether this device improves clinical outcomes in hyperkyphosis.

## ABBREVIATIONS

AISI: American Iron and Steel Institute
MDC: minimal detectable change
TKA: thoracic kyphosis angle
